# Retraction: Nutritional and Biochemical Parameters Among Multiple Sclerosis Patients: A Case-Control Study

**DOI:** 10.7759/cureus.r31

**Published:** 2021-05-27

**Authors:** Asjad Salman, Sabeeh Islam, Muhammad Awais Saleh, Khurram Khaliq Bhinder, Zainab Malik, Iqra Tahir, Muhammad A Naveed

**Affiliations:** 1 Internal Medicine, Punjab Rangers Teaching Hospital, Lahore, PAK; 2 Internal Medicine, St. Vincent Health Center, Buffalo, USA; 3 Internal Medicine, Government Khawaja Muhammad Safdar Medical College and Allied Institutions, Sialkot, PAK; 4 Radiology, Shifa International Hospital Islamabad, Islamabad, PAK; 5 Internal Medicine, Lahore General Hospital, Lahore, PAK; 6 Cardiology, Allama Iqbal Memorial Teaching Hospital, Sialkot, PAK; 7 Cardiology, Government Khawaja Muhammad Safdar Medical College and Allied Institutions, Sialkot, PAK

This article has been retracted due to allegations that the study data was plagiarized from an already-published article in Neurology India: https://www.neurologyindia.com/article.asp?issn=0028-3886;year=2020;volume=68;issue=4;spage=867;epage=874;aulast=Akbay

After conducting our own investigation, Cureus has made the decision to formally retract this article. 

A key condition of article submission is that authors must explicitly declare that their work is original and has not appeared in publication elsewhere. Re-use of any data must be appropriately cited. As such this article represents a severe abuse of the scientific publishing system. The Cureus Journal of Medical Science takes a very strong view on this matter and we apologize that this was not detected during the submission process. 

 

